# Transcriptomic Profile of Early Antral Follicles: Predictive Somatic Gene Markers of Oocyte Maturation Outcome

**DOI:** 10.3390/cells14100704

**Published:** 2025-05-12

**Authors:** Alessia Peserico, Barbara Barboni, Chiara Camerano Spelta Rapini, Chiara Di Berardino, Giulia Capacchietti, Angelo Canciello, Fani Konstantinidou, Marisa Donato, Liborio Stuppia, Valentina Gatta

**Affiliations:** 1 Department of Bioscience and Technology for Food, Agriculture and Environment, University of Teramo, 64100 Teramo, Italy; apeserico@unite.it (A.P.); bbarboni@unite.it (B.B.); ccameranosr@unite.it (C.C.S.R.); gcapacchietti@unite.it (G.C.); acanciello@unite.it (A.C.); mdonato1@unite.it (M.D.); 2 Department of Neuroscience, Imaging and Clinical Sciences, School of Medicine and Health Sciences, “G. d’Annunzio” University of Chieti-Pescara, 66100 Chieti, Italy; fani.konstantinidou@unich.it (F.K.); liborio.stuppia@unich.it (L.S.); v.gatta@unich.it (V.G.); 3 Unit of Molecular Genetics, Center for Advanced Studies and Technology (CAST), “G. d’Annunzio” University of Chieti-Pescara, 66100 Chieti, Italy

**Keywords:** follicle biomarkers, oocyte quality, follicle-enclosed oocyte maturation, IVF, transcriptomic profile, driver genes

## Abstract

Early antral follicles (EAfs) offer oocyte potential in Assisted Reproductive Technology (ART), but most fail to mature under current in vitro maturation (IVM) protocols. This study examines transcriptomic profiles of the follicular wall (FW) compartment during IVM in ovine EAfs using a 3D follicle-enclosed oocyte (FEO) culture to identify somatic gene markers predicting oocyte maturation success. Differentially expressed genes (DEGs) were identified across three comparisons: pre- vs. post-hCG in FW enclosing mature/fertilizable (1) or immature (2) oocytes, and post-hCG between FW supporting successful vs. failed maturation (3). Network analysis highlighted key modulated and HUB genes. Two DEG categories emerged: genes regulating meiosis resumption and genes defining follicular signatures linked to oocyte competence. Meiosis resumption involved ECM remodeling, hypoxia, and relaxin signaling activation, while proliferative and metabolic pathways were downregulated. *MMP13* and *EGFR* regulated the ECM pathway, working for meiosis resumption, while *TGFB1* predicted failure. Oocyte competence involves ECM activation and the suppression of stress and cell cycle pathways, with *ITIH4* being conducive to central HUB tuning inflammation and angiogenesis-dependent maturation. This study reveals molecular mechanisms behind follicle maturation, identifying transcriptomic signatures for FW releasing mature/fertilizable and incompetent oocytes. It confirms known biomarkers and uncovers new regulators, offering tools to assess follicle quality, improve IVF–oocyte selection, and enhance fertility preservation.

## 1. Introduction

Assisted Reproductive Technologies (ARTs) have advanced significantly, addressing infertility in humans and animals. However, reliance on small and medium antral follicles limits oocyte availability. In this context, early antral follicles (EAfs), more abundant than antral follicles, emerge as an underused reserve that, with optimized maturation, could expand the pool of fertilizable gametes for reproductive applications [1,2,3,4,5].

EAfs, naturally present in ovaries, can also be generated in vitro as the final stage of folliculogenesis protocols across species (mouse: [6,7]; sheep: [2,4,8,9,10,11]; bovine: [12]; buffalo: [13]; goat: [14,15,16,17]). These follicles can be cultured from preantral stages (PAfs) or earlier follicular phases, expanding the gamete pool for embryo production. This approach enhances fertility preservation and reproductive engineering, offering innovative strategies to improve ART [18,19,20,21].

EAfs house oocytes undergo key developmental processes [4,22], including epigenetic remodeling [4,23], chromatin modifications [2,22,24], and telomere regulation [25], critical for oocyte quality and embryonic development [4]. The transition from late PAfs to EAfs marks a crucial phase where oocytes complete growth and acquire functional specializations. Advances in in vitro Folliculogenesis and animal studies highlight the promise of EAfs in ART, paving the way for clinical applications [1,2,19,26,27,28,29,30].

Despite their potential, oocytes from EAfs often show lower developmental competence compared to those from advanced-stage follicles, requiring specialized in vitro maturation (IVM) protocols. Traditional IVM targets cumulus–oocyte complexes (COCs) and has been extensively optimized in cattle [31,32,33] and sheep [9,26,34,35]. As an alternative, the follicle-enclosed oocyte (FEO) model exploits the intrinsic gonadotropin sensitivity of EAfs to achieve controlled maturation. This approach has been tested in hamsters [36], rats [37], rabbits [38], sheep [1,39,40], and pigs [41].

Recent studies show that bioinspired 3D culture systems markedly improve the maturation of oocytes derived from EAfs, outperforming conventional COC-IVM outcomes [1,2].

Beyond refining IVM techniques, advancements in ART are also focusing on ovarian stroma biomaterials and tissue culture methodologies to increase the availability of EAfs through in vitro Folliculogenesis [42,43,44,45,46,47]. These approaches hold great promise for optimizing the recruitment of fertilizable gametes, expanding the oocyte pool, and enhancing the efficiency of ART applications [2,48,49].

Notably, human chorionic gonadotropin (hCG) is crucial for oocyte maturation in EAfs because of its LH-like activity and minor residual FSH-like effects within the FEO system [1,2]. Experimental evidence shows that EAf-derived oocytes do not progress beyond meiotic arrest unless exposed to hCG [1]. Among the available hormonal stimuli, hCG provides the most effective and reliable maturation trigger in medium-sized mammalian models, supplying adequate gonadotropic support without supplemental FSH [1,2]. Nevertheless, only a small proportion of EAf-derived oocytes reach full competence, underscoring the need to clarify the factors determining success or failure in response to hCG [1].

Compelling evidence demonstrates that the follicular wall (FW)—mural granulosa plus theca—is both a sensor and effector of the preovulatory LH/hCG surge. Moreover, the gonadotrophin elicits a rapid, genome-wide transcriptional reset in these somatic layers that licenses meiotic resumption and, ultimately, shapes embryo potential [50,51,52,53,54,55,56,57]. Cross-species granulosa–oocyte co-profiling further shows that somatic gene signatures faithfully mirror oocyte competence, positioning the follicular transcriptome as a robust surrogate endpoint [58,59,60,61,62,63]. Exploiting this read-out is minimally invasive and promises novel biomarkers, although inter-species and culture-specific variability still demands systematic validation.

Against this backdrop, we employed the FEO-IVM model, which recreates physiological maturation (meiotic resumption occurs solely in response to a controlled LH/hCG stimulus), providing a clean link between somatic transcriptional dynamics and definitive oocyte fate. Using ovine early antral follicles (EAfs), we profiled the FW transcriptome pre- and post-hCG and contrasted EAfs that produced metaphase-II, fertilisable oocytes with those whose oocytes remained at the germinal vesicle stage. Differentially expressed genes (DEGs) associated with follicular activation and oocyte maturation were then prioritized as candidate biomarkers. By coupling this strategy to the FEO-IVM platform, the present study aims to refine IVM protocols, nominate non-invasive somatic markers of oocyte quality, and, ultimately, enhance ART approaches for fertility preservation and reproductive medicine.

## 2. Materials and Methods

### 2.1. Ovary Collection

The study involved collecting ovaries from Appenninica sheep lambs from discarded tissues from a local slaughterhouse. These ovaries, from prepubertal sheep around 5 months of age, intended for meat production, were obtained from a local slaughterhouse. They were then transported to the laboratory in a temperature-controlled container to ensure stability during the journey, which typically took less than an hour. Upon arrival, the ovaries were rinsed multiple times with a 0.9% NaCl solution, supplemented with 1 mg/mL Benzoxonium chloride (Bialcol #032186013, Vemedia Pharma, Nola, NA, Italy). After the medulla was removed, the ovaries were placed in a HEPES-buffered TCM199 medium (#M7528, Sigma Aldrich, St. Louis, MO, USA) and cut into uniform cortical fragments approximately 0.5 × 0.5 × 0.5 cm in size.

### 2.2. Ovarian Surface Epithelium (OSE) Cell Collection for Follicle-Enclosed Oocyte (FEO) Coculture System

Ovarian surface epithelial (OSE) cells were extracted from the ovarian cortex of prepubertal ovaries using a surgical scalpel. Prior to extraction, the ovaries were incubated in a 0.25% Trypsin/EDTA solution (200 mg/L) at 38.5 °C for 5 min. The resulting cell suspensions were transferred into a 6 cm petri dish containing DPBS solution with 30% fetal bovine serum (FBS; #10270-106, Gibco, Thermo Fisher Scientific, Waltham, MA, USA) to neutralize the Trypsin (#25200056, Gibco, Thermo Fisher Scientific, Waltham, MA, USA). After centrifugation, the supernatant was discarded. The OSE cells were then seeded into a growth medium consisting of alphaMEM (#BE02-002F, Lonza, Basel, CH, Switzerland), 20% fetal bovine serum (FBS: #10270-106, Gibco, Thermo Fisher Scientific, Waltham, MA, USA), 1% glutamine (#BE17-605E/U1, Lonza, Basel, CH, Switzerland), and antibiotics including 75 mg/L of penicillin-G and 50 mg/L of streptomycin sulfate (#DE17-602E, Lonza, Basel, CH, Switzerland) for one passage of cell expansion. Once expanded, the OSE cells were seeded into the bottom of the transwell system to create a feeder cell monolayer for FEO coculture.

### 2.3. FEO In Vitro Maturation from EAfs

Ovine EAfs were manually isolated from cortical fragments using sterile 32 G needles under a stereomicroscope in a laminar flow hood. The selection of EAfs was based on their size and morphology to ensure that the theca layer remained intact. For FEO maturation experiments, the selected EAfs follicles had an average diameter of 360 ± 31 μm. Before incubation, each EAfs was carefully examined using an inverted-phase microscope equipped with time-lapse imaging software (NIS-Elements Advanced Research, Version 4.51.00, Eclipse Ti Series, Nikon Europe BV, Amstelveen, The Netherlands) to measure the diameter and assess for any morphological signs of degeneration. This included checking for the darkening of somatic cells or oocytes, loss of compactness in the granulosa cell layer, or any disruption of the basal membrane. The selected EAfs were placed in transwell culture systems, each containing 96-well plates with U-shaped wells. The follicles were placed on scaffold holders made of PCL-patterned electrospun materials, as previously described [9], while the bottom of the transwell was lined with a monolayer of OSE cells.

Each transwell system was filled with 100 μL of maturation medium, composed of alphaMEM (Cat. No. BE02-002F Lonza, Basel, CH, Switzerland), 20% FBS (#10270-106, Gibco, Thermo Fisher Scientific, Waltham, MA, USA), 1% glutamine (#BE17-605E/U1, Lonza, Basel, CH, Switzerland), and antibiotics (75 mg/L penicillin-G and 50 mg/L streptomycin sulfate; #DE17-602E, Lonza, Basel, CH, Switzerland). The medium was also supplemented with 25 IU/mL human chorionic gonadotropin (hCG; equivalent to 6 µg/mL Chorulon^®^, MSD Animal Health, Segrate, MI, Italy) to promote in vitro maturation. The EAfs were incubated in this maturation medium for 24 h at 38.5 °C with 5% CO_2_. At the end of the FEO maturation process, the follicles were opened, and the FW were separated from the oocyte. These cells were then grouped according to the nuclear stage of the oocyte for further analysis and collection. Additionally, FW from EAfs were collected before the addition of hCG and separated from their respective oocytes, forming the control group of FW from EAfs prior to gonadotropic stimulation (FWs enclosing oocytes in the GV stage).

### 2.4. Oocyte Nuclear Stage Assessment and FW Collection

Oocytes obtained from FEO in vitro maturation were stripped of their surrounding cumulus cells, stained with DAPI, and visualized using the time-lapse imaging software NIS-Elements (Eclipse Ti Series, Nikon, Tokyo, Japan) to assess the nuclear stage. The oocytes were categorized into different meiotic nuclear stages, including germinal vesicle (GV) and Metaphase II (MII), as described previously [4].

### 2.5. In Vitro Embryo Production

The developmental potential of MII oocytes was assessed through in vitro fertilization (IVF). IVF was conducted using frozen-thawed semen obtained from rams with proven fertility, as described previously [4]. In summary, fertilization was performed in 50 µL drops with a sperm concentration of 1 × 10^6^ sperm/mL at 38.5 °C, in a humidified environment of 5% CO_2_ in air, for 20–22 h. After 2 and 8 days of culture, the uncleaved and cleaved embryos were examined under an inverted microscope with time-lapse imaging software (NIS-Elements Advanced Research, Version 4.51.00, NIS-Elements, Eclipse Ti Series, Nikon, Japan) to assess developmental stages by counting the number of blastomeres. The fertilization rate was determined by the number of cleaved embryos on day 2, and the blastocyst rate was calculated on day 8 based on the cleaved embryos recorded on day 2.

### 2.6. Microarray Transcriptomic Analysis

Total RNA was extracted from pools of five follicular walls for each experimental group—namely, GV-stage FWs before hCG stimulation, GV-stage FWs after hCG treatment (unsuccessful), and FWs from MII oocytes that developed to the blastocyst stage (successful) upon IVF—using the Single-Cell RNA Purification Kit (#51800, Norgen Biotek Corp., Thorold, ON, Canada) according to the manufacturer’s protocol. The transcriptomic profile analysis was carried out using the MicroArray GeneChip System (Applied Biosystems, Thermo Fisher Scientific, Waltham, MA, USA).

More specifically, microarray analysis was conducted on all FW samples using the GeneChip WT PLUS Reagent kit (Applied Biosystems, Thermo Fisher Scientific, Waltham, MA, USA). A concentration of 100 ng of total RNA from each sample was used as starting material to synthesize complementary DNA (cDNA). Complementary RNA (cRNA) was subsequently synthesized from in vitro transcription of cDNA and then purified and reverse transcribed. Finally, single-stranded cDNA (ss-cDNA) was synthesized, purified, fragmented, and labeled following the manufacturer’s instructions. Array Hybridization was performed using the GeneChip™ Hybridization, Wash, and Stain Kits and the OviGene 1.0 ST GeneChip Arrays (Ovine; Applied Biosystems) on the GeneChip Hybridization Oven 645 and GeneChip Fluidics Station (Applied Biosystems, Thermo Fisher Scientific, Waltham, MA, USA). Scanning was performed using the GeneChip Scanner 3000 7G and Affymetrix Command Console (AGCC) software, Version AGCC 7.0 (Applied Biosystems, Thermo Fisher Scientific, Waltham, MA, USA). The raw image data obtained after scanning were analyzed using the Transcriptome Analysis Console (TAC) software (ver. 4.0.2, Applied Biosystems). The raw data were normalized following the signal space transformation robust multi-chip analysis (SST-RMA) algorithm. The detected above-background (DABG) cutoff by default was set to 0.05. The positive versus negative area under the curve (AUC) value was set greater than or equal to 0.7. Finally, genes that passed the filter criteria of *p* value < 0.05 (one-way between-subjects ANOVA) and fold change > 2 for upregulation and <−2 for downregulation were considered as differentially expressed genes (DEGs).

### 2.7. Network Creation, Visualization, and Analysis

Three datasets (referred to, respectively, as pairwise 1, 2, and 3) were generated using the Transcriptome Analysis Console (TAC) 4.0 Software (Applied Biosystems, Thermo Fisher Scientific, Waltham, MA, USA). Each dataset was used as input to build a network using the Cytoscape 3.9.1 software (http://www.cytoscape.org, accessed on 2 May 2024). Each network was then analyzed with the dedicated plug-in Network Analyzer of Cytoscape according to a previous report [5,64] by computing the following topological parameters: number of nodes, number of edges, the average number of neighbors, network diameter, characteristic path length, clustering coefficient, and connected components (Supplementary Materials File S1), Additional analyses applied to networks and nodes have been listed in the following sub-sections.

### 2.8. Identification of Highly Interconnected Regions (Modules) Using the MCODE Algorithm

The Cytoscape plugin Molecular Complex Detection (MCODE) [65] was utilized to identify and analyze clusters of densely interconnected nodes (modules) within the network. Modules with k-core values greater than 5 and node degrees exceeding 5 were selected for further KEGG pathway analysis, which was conducted using the Cytoscape ClueGO plugin. The reference database used for network construction and pathway analysis was *Ovis aries* (Taxid:9940).

### 2.9. Identification of Drivers Within Network Modules

The study distinguished two types of drivers: highly modulated DEGs and highly interconnected DEGs (HUBs). Highly modulated DEGs, identified as outlier genes based on their expression levels, were detected by comparing gene expression values applying the interquartile range method [66,67] and visualized using a volcano plot (GraphPad Prism 10.1.1; https://www.graphpad.com/, accessed on 2 May 2024). Of note, a significance threshold of *p* < 0.05 was applied to determine whether a gene’s expression significantly deviated from the expected Gaussian distribution of non-outliers.

HUBs were identified using the CytoHubba plugin in Cytoscape [68] by following a four-step workflow according to recent literature methodological evidence [69,70,71,72,73,74,75].

First, 12 centrality coefficients supported by CytoHubba (Supplementary Materials File S2) were calculated for each DEG, providing each DEG with a score, and plotted using PCA (from GraphPad 10.1.1 Prism; https://www.graphpad.com/, accessed on 2 May 2024).

DEGs were then ranked based on the scores of these centrality coefficients (II).

The top 10 DEGs for each centrality coefficient (those with the highest scores) were selected (III), and DEGs appearing in the top 10 for at least 5 of the 12 algorithms were identified as hubs (IV).

### 2.10. Venn Diagram

A Venn diagram tool (https://bioinformatics.psb.ugent.be/webtools/Venn/, accessed on 2 May 2024) was used to illustrate the shared and signature DEGs across the networks.

### 2.11. Microarray Validation Through Real-Time qPCR

The total RNA was extracted with a Single-Cell RNA Purification Kit (#51800, Norgen Biotek Corp., Thorold, ON, Canada) following the manufacturer’s instructions. A total of 1 μg of total RNA was retrotranscribed using oligodT primers (Bioline, Memphis, TN, USA) and Tetro Reverse Transcriptase (Bioline, Bioline, Memphis, TN, USA), following the manufacturer’s instructions. The qPCRs were carried out in triplicate using the SensiFAST SYBR Lo-ROX kit (Bioline, Bioline, Memphis, TN, USA) on a QuantStudio3 System (Life Technologies, Thermo Fisher Scientific, Carlsbad, CA, USA), according to the manufacturer’s instructions. The following PCR conditions were used for all the experiments: 95 °C for 10 min, followed by 40 cycles at 95 °C for 10 s and 60 °C for 30 s. Relative quantification was performed by using the ∆∆Ct method. Glyceraldehyde 3-phosphate dehydrogenase (*GAPDH*) and Tyrosine 3-Monooxygenase/Tryptophan 5-Monooxygenase Activation Protein Zeta (*YWHAZ*) were selected from among the housekeeping genes for gene quantification. The expression profiles were similar for both reference genes. The primer sequences are reported in Supplementary Materials File S3.

### 2.12. Statistical Analysis

Three independent biological replicates for each experimental group (GV start-point, MII end-point, and GV end-point) were conducted. The statistical analysis was conducted as follows:

The Microarray GeneChip assays were performed through a One-Way ANOVA, followed by an empirical Bayes correction for differential expression using the Transcriptome Analysis Console (TAC software; version 4.0.1, Thermo Fisher Scientific, Waltham, MA, USA).

For the analyses related to oocyte maturation performance, embryonic developmental competence, and qPCR validation, One-Way ANOVA was performed with GraphPad Prism 10.1.1 (https://www.graphpad.com/, accessed on 2 May 2024), with *p*-values less than 0.05 considered statistically significant.

For the ClueGO analysis of the MCODE-defined modules, *p*-values < 0.05 were deemed significant and corrected with the Bonferroni test.

## 3. Results

### 3.1. Comparative Transcriptomic Analysis of FW Compartment from EAfs Enclosing Competent and Incompetent Oocytes

This study profiles, within EAfs, the transcriptome of the FW compartment—the combined mural granulosa and theca cell layers—to elucidate the hCG-driven signaling events that promote oocyte maturation.

EAfs were selected as a source of gonadotropin-sensitive follicles, which, through advanced IVM protocols, can yield an additional pool of fully grown oocytes for ARTs. The validated FEO-IVM method employs a 3D culture system using PCL-patterned scaffolds, significantly enhancing the number of fertilizable oocytes from EAfs.

As shown in Table 1A, FEO-IVM maintains meiotic arrest without hormonal stimulation (100% GV) and supports meiosis resumption in most oocytes (89% GVBD+MII) upon hCG exposure. Of the matured MII oocytes, 58.2% were fertilized, though only ~10% progressed to advanced embryonic stages (Table 1B). The small subset of EAf-enclosed oocytes that failed to initiate maturation (GV 11%) in response to hormonal stimulation provided the FWs used to analyze the transcriptomic profile of structures incapable of supporting oocyte maturation.

**Figure 1:**
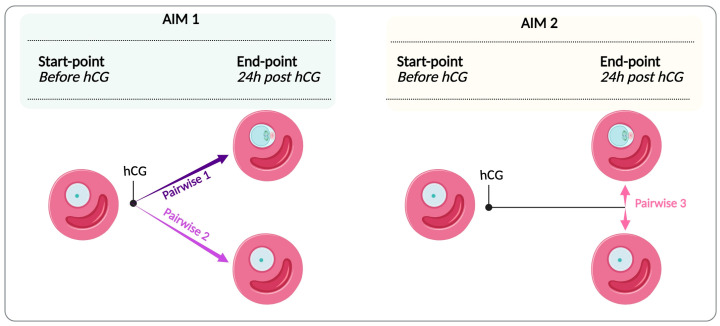
Experimental transcriptome plan. FEO IVM was induced by hCG to isolate FWs from EAfs associated with oocytes that reached MII or failed to resume meiosis, thus remaining at the GV stage. This setup supported two aims: AIM 1 (light green), comparing FW transcriptomes ±hCG with different maturation outcomes (MII or GV end-point vs. GV start-point); AIM 2 (light yellow), comparing FWs from successful and unsuccessful EAfs post-hCG (MII end-point vs. GV end-point). Created in BioRender., Peserico, A. (2025), https://www.biorender.com/.

The experimental design (Figure 1) compared follicular cells (FWs) from EAfs under three conditions: (i) EAfs collected before hCG stimulation (GV start-point); (ii) EAfs whose oocytes progressed to the metaphase-II (MII) stage after hCG—designated successful EAfs (MII end-point); and (iii) EAfs whose oocytes failed to resume meiosis and remained at the germinal vesicle (GV) stage despite hCG—designated unsuccessful EAfs (GV end-point).

Each group was interrogated with an ovine whole-transcriptome microarray. For each pairwise comparison, differentially expressed genes (DEGs) were first delineated and subsequently subjected to network-based prioritization to extract putative driver genes. The pairwise analyses addressed two biological objectives: AIM 1—to elucidate follicular-cell driver genes governing the hCG-dependent activation of EAfs. AIM 2—to identify candidate drivers that discriminate follicular cells from EAfs capable of, versus refractory to, hCG-induced oocyte meiotic maturation. For AIM 1, two pairwise comparisons were conducted: first, FWs from successful EAfs releasing MII oocytes (MII end-point) versus unstimulated controls (GV start-point); second, FWs from unsuccessful EAfs releasing GV oocytes (GV end-point) versus GV start-point as control. AIM 2 involved a third pairwise comparison between FWs from successful and unsuccessful maturation outcomes (MII vs. GV end-point) to highlight transcriptomic differences associated with divergent oocyte fates following hCG treatment.

In Table 2, DEGs obtained from three pairwise comparisons after the analysis of 22,141 *Ovis aries* genes were summarized, adopting a fold change greater than 2 and a *p*-value less than 0.05.

In more detail, most DEGs were downregulated across the three pairwise comparisons—66%, 56%, and 90.7%, respectively.

### 3.2. FW Driver Genes Promoting Maturation in EAfs

#### 3.2.1. Network 1_MII-GV_ and Network 2_GV-GV_ Signaling Modules

To identify the gene markers that characterized FW supporting hCG-induced meiotic resumption, the DEGs identified in pairwise 1 (end-point MII vs. start-point GV: _MII-GV_) and pairwise 2 (end-point GV vs. start-point GV: _GV-GV_) comparisons were processed using Cytoscape version 3.9.1. This procedure generated two networks representing, respectively, successful (Network 1_MII-GV_) and unsuccessful (Network 2_GV-GV_) EAfs.

Genes with uncharacterized function and long non-coding RNAs were excluded from the generation of the 2 Networks since they are not recognized by Cytoscape (457 of Network 1_MII-GV_ and 437 of Network 2_GV-GV_ were not recognized).

More in detail, Network 1_MII-GV_ comprised 2.071 nodes (annotated ovine DEGs), 15.712 edges, and 92 connected components. Network 2_GV-GV_ recognized fewer nodes and edges (1.562 and 6.546, respectively) yet retained a similar number of connected components (n = 95). Topological metrics confirmed the scale-free nature of both the networks (Supplementary Materials File S4).

**Scheme 1:**
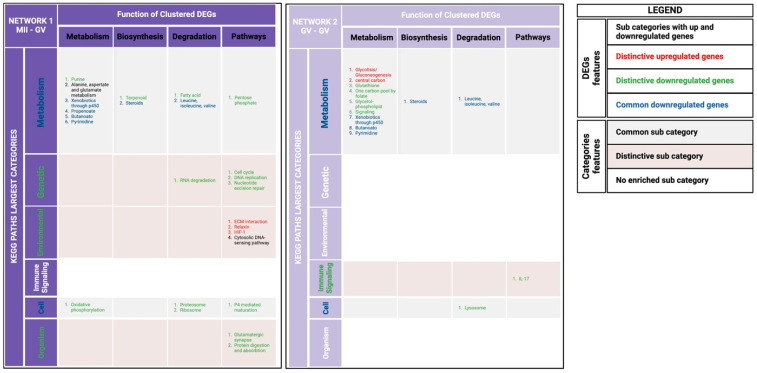
Network 1_MII-GV_ and Network 2_GV-GV_ DEGs modules identified with the MCODE Cytoscape plugin and annotated using ClueGO.

The networks were analyzed using the MCODE clustering algorithm to extract highly interconnected clusters (modules) of DEGs (Supplementary Materials File S5). The resulting modules were functionally annotated in ClueGO and consolidated into the six broadest KEGG pathway categories. Specifically, Network1_MII-GV_ recognized 23.3% (482/2071) of DEGs clustered into 10 functional modules belonging to five out of the seven largest KEGG categories (Scheme 1).

Network 1_MII-GV_ denotes the network built from pairwise 1, whereas Network 2_GV-GV_ corresponds to the network generated from pairwise 2. Modules were extracted with MCODE (k-core ≥ 5, node degree > 5; see Section 2.8) and functionally annotated in ClueGO; resulting terms were consolidated into the major KEGG pathway categories. Full statistics and thresholds are provided in Supplementary Materials File S5. The color code distinguishes (i) shared vs. unique DEGs and (ii) the different KEGG categories, while symbol style denotes up- or downregulation.

Three categories were distinctive of Network 1_MII-GV_: genetic information processing, environmental information processing, and the organismal system. The environmental information processing category recognized mainly upregulated DEGs (red signed genes) involved in ECM remodeling, relaxin, HIF signaling, and cytosolic DNA sensing pathways (modules 7–8). Conversely, the genetic information processing KEGG categories exclusively comprised downregulated DEGs (green signed genes) mapped to RNA and cell cycle pathways (modules 2 and 3).

Metabolism was the most populated KEGG path and the largest category of Network 1_MII-GV._ It consisted mainly of downregulated DEGs related to fatty acid and amino acid degradation (both in modules 3 and 6), terpenoid/steroid biosynthesis (module 3), and two distinctive metabolic pathways (purine metabolism in module 5 and pentose phosphate in module 7). Within cellular processes, additional downregulated DEGs involved in progesterone-mediated maturation (modules 2 and 3), oxidative phosphorylation (module 1), and lysosome and proteosome processes (module 1 and 5, respectively).

Network 2_GV-GV_ recognized a lower percentage of clustered DEGs (13.6%: 213 out of 1562) that belonged to six diverse modules grouped in three of KEGG’s largest categories (Scheme 1). A distinctive feature was the immune signaling and response (modules 2, 6). It was composed of downregulated DEGs, all involved in the IL-17 pathway (module 2), and of genes with a mixed profile of expression (signed in black) related to viral protein interactions with cytokine and cytokine receptor pathways.

As in Network 1_MII-GV_, metabolism was also the most enriched KEGG category in Network 2_GV-GV_; however, the pathway composition differed. Upregulated genes mapped to glycolysis/gluconeogenesis (module 1) and carbon metabolism (module 3), whereas downregulated genes populated glutathione, pyrimidine, one-carbon-pool–by-folate (module 4), and various signaling-metabolism pathways (module 5).

#### 3.2.2. Driver DEGs of Network 1_MII-GV_ and Network 2_GV-GV_

The MCODE-derived clusters were further prioritized by applying two complementary filters. First, highly modulated DEGs—those exhibiting the greatest fold-changes and thus representing the most immediate, co-regulated response to hCG—were delineated. Second, highly interconnected DEGs (HUBs) were identified based on their central topological indices, indicating a pivotal role in organizing network behavior and governing the meiotic maturation program. This integrated approach distinguishes transcripts that respond most vigorously to hCG from those that structurally orchestrate the underlying signaling circuitry.

##### Highly Modulated DEGs of Network 1_MII-GV_ and Network 2_GV-GV_

Highly modulated DEGs were visually selected using a volcano plot, which enabled their stratification based on gene modulation levels and significance in the two networks.

**Figure 2:**
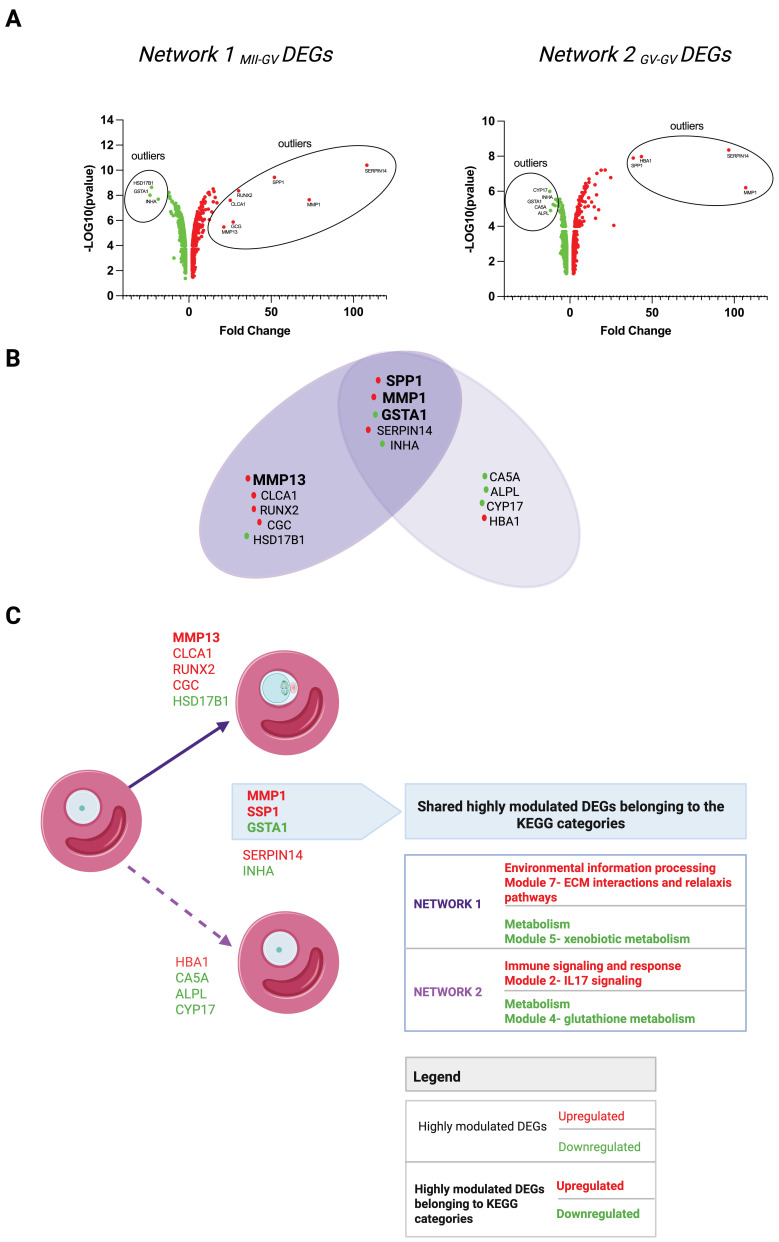
Identification of highly modulated genes characterizing the FW of successful and unsuccessful EAfs: (**A**) Volcano plot showing the upregulated (red) and downregulated (green) DEGs in Network 1_MII-GV_ (EAfs releasing competent oocytes) and Network 2_GV-GV_ (EAfs releasing incompetent oocytes). DEGs exhibiting significantly high levels of modulation (outliers) are enclosed in a circle. Highly modulated DEGs were recorded through MCODE as unclustered, since, outside of the highly connected clusters, they were not selected as critical nodes, even if they met the criteria outlined in point (I). (**B**) Venn diagram of the outlier DEGs between the two networks, illustrating common DEGs as well as distinctive ones. Furthermore, the DEGs populating the highly interconnected modules in the respective networks are highlighted in bold. (**C**) Graphical representation of the highly modulated DEGs, highlighting the common and distinctive ones. DEGs within the highly interconnected KEGG modules in their respective networks are shown in bold, whereas highly modulated DEGs outside of the KEGG categories are displayed in regular font. Shared, highly modulated DEGs from KEGG categories are enclosed in the light blue box. The corresponding modules are specified in the legend. Created in Biorender. Peserico, A. (2025), https://www.biorender.com/.

As summarized in Figure 2A, the highly modulated DEGs (outlier DEGs) are as follows:

− Network 1_MII-GV_: Seven upregulated (*MMP1*, *SPP1*, *CLCA1*, *MMP13*, *SERPIN14*, *GCG*, *RUNX2*) and three downregulated (*GSTA1*, *HSD17B*, *INHA*);
− Network 2_GV-GV_: Four upregulated (*MMP1*, *SPP1*, *HBA1*, *SERPIN14*) and five downregulated (*GSTA1*, *CYP17*, *ALPL*, *CA5A*, *INHA*).

The highly modulated DEGs identified using MCODE but positioned outside densely connected clusters (unclustered) were not regarded as critical nodes, even though they met the criteria outlined in point (I). Consequently, within Network 1_MII-GV_, only three out of seven upregulated (*MMP1*, *SPP1*, *MMP13*) and one out of three downregulated (*GSTA1*) outliers fell inside the MCODE modules (Scheme 1). All three upregulated genes resided in module 7 (environmental information processing), which encompasses the ECM–receptor interaction and relaxin-signaling pathways, whereas the single downregulated gene was located in module 5 (Metabolism), linked to xenobiotic metabolism.

Of note, *MMP13* emerged as the only distinctive upregulated outlier with a key role in hCG-driven maturation within the FW (Network 1_MII-GV_). It displayed a great modulatory function, being linked with several other upregulated DEGs in the ECM remodeling pathway (module 7 of environmental information processing, the KEGG path’s largest category), such as *BMP2*, *CD44*, *CXCR4*, *MMP1*, *MMP9*, *PLAU*, *SPP1*, and *TIMP3* (Supplementary Materials File S6).

In Network 2_GV-GV_, two out of four upregulated (*MMP1*, *SPP1*) and one out of five downregulated (*GSTA1*) DEGs were located within MCODE modules. Although Venn analysis flagged these DEGs as common outliers to both networks (Figure 2B), the MCODE approach revealed network-specific functions: in Network 2_GV-GV_, the upregulated outliers operated within the IL-17 signaling (module 2 of Immune signaling and response KEGG category), whereas the downregulated *GSTA1*, clustered into module 5 (metabolism KEGG category,) implicated glutathione metabolism signaling.

A comprehensive overview of the highly modulated genes unique to, or shared between, the two networks is provided in Figure 2C.

##### HUBs of Network 1_MII-GV_ and Network 2_GV-GV_

**Figure 3:**
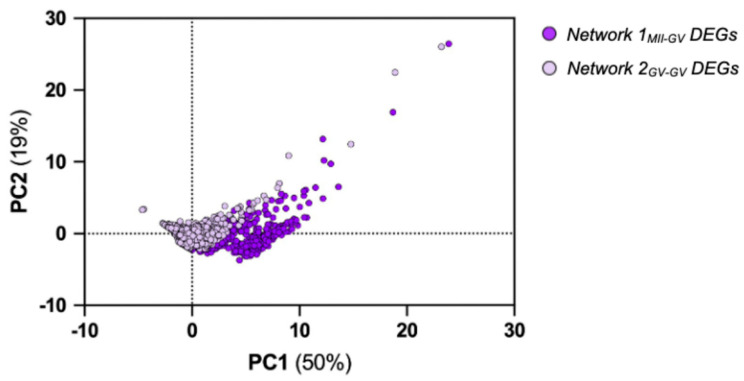
Principal component analysis (PCA) demonstrates a sharp separation between the DEGs belonging to FW enclosing MII and those enclosing GV oocytes.

Finally, a computational workflow was applied to identify HUBs, DEGs that preserve network architecture and govern its functionality. In detail, HUBs were detected with the CytoHUBba plugin in Cytoscape via a four-step procedure. In more detail, through step I, each DEG was scored for each centrality categories (Closeness, Degree, Maximal Clique Centrality, Radiality, Stress, Maximum Neighborhood Component, Density of Maximum Neighborhood Component, Betweenness, Clustering Coefficient, EcCentricity, BottleNeck, and Edge Percolated Component) to measure the network influence through the relative coefficient. Once each coefficient of centrality was defined in step II, the gathered scores were defined through principal component analysis (PCA) using the multivariate approach. The analysis enabled the detection of the differences between the two networks and reduced the dataset’s complexity into two principal components. These components explained approximately 70% of the total variance (Figure 3; see also Supplementary Materials File S7).

DEGs of each network were then filtered for their priority, and the top 10 were ranked based on their centrality coefficient scores (step III). Finally, to obtain a reliable prediction of DEGs with a HUB role, only DEGs ranked as top 10 for at least 5 of the 12 centrality coefficient scores selected (step IV; Supplementary Materials File S8). To distinguish between shared or distinctive HUBs, Venn diagram analysis was conducted (Supplementary Materials File S8).

The computational analysis identified seven HUBs in Network 1_MII-GV_ (Supplementary Materials File S8, sheet 3). *EGFR* was upregulated, whereas the others were downregulated and mapped to functions in DNA transcription (*POLR2C*, *POLR2F*), cell cycle regulation (*CDCA8*), amino acid biosynthesis (*ALDH18A1*), and mitochondrial protein synthesis (*MRPL2*, *MRPS12*).

Analogously, seven HUBs were detected in Network 2_GV-GV_ (Supplementary Materials File S8, sheet 4). Only *TGFB1 and MET* were upregulated, both linked to cell growth, motility, and morphogenesis-related pathways. The downregulated HUBs were involved in cAMP-dependent signaling (*PRKACA*, *PRKACB*), immune response protein (*ISG15*), DNA transcription enzyme (*POLR2E*), and cell adhesion and migration antigen (*CD44*).

Venn diagram analysis revealed a unique shared HUB between the two networks: *TP53*, which was downregulated and is implicated in DNA damage repair.

Overall, the gene driver sets for each network are comprised of the following:

Network 1_MII-GV_: One highly modulated DEG (MMP13) and seven HUBs (POLR2C, POLR2F, CDCA8, ALDH18A1, MRPL2, MRPS12, and EGFR).

Network 2_GV-GV_: Seven HUBs (TGFB1, MET, PRKACA, PRKACB, ISG15, POLR2E, and CD44).

### 3.3. Signature Genes Distinguishing FWs from Successful Versus Unsuccessful EAfs at the End of the Maturation Phase

Following the identification of the key FW genes triggering oocyte maturation, the study then turned to those genes whose expression profiles distinguish follicular signatures predictive of oocyte competence at the maturation end-point (AIM 2 Figure 1).

To this end, the following workflow was applied to filter DEGs in the EA FW sampled 24 h after hCG stimulation.

(I) Analyze the transcriptomic landscape of FW enclosing MII (successful maturation) and GV (unsuccessful maturation) at the end-point of FEO in vitro maturation (pairwise 3 comparison).

(II) Build the Network 3_MII-GV end-point_ using the DEGs identified from pairwise 3 as inputs.

(III) Classify DEGs according to their functional role.

**Figure 4:**
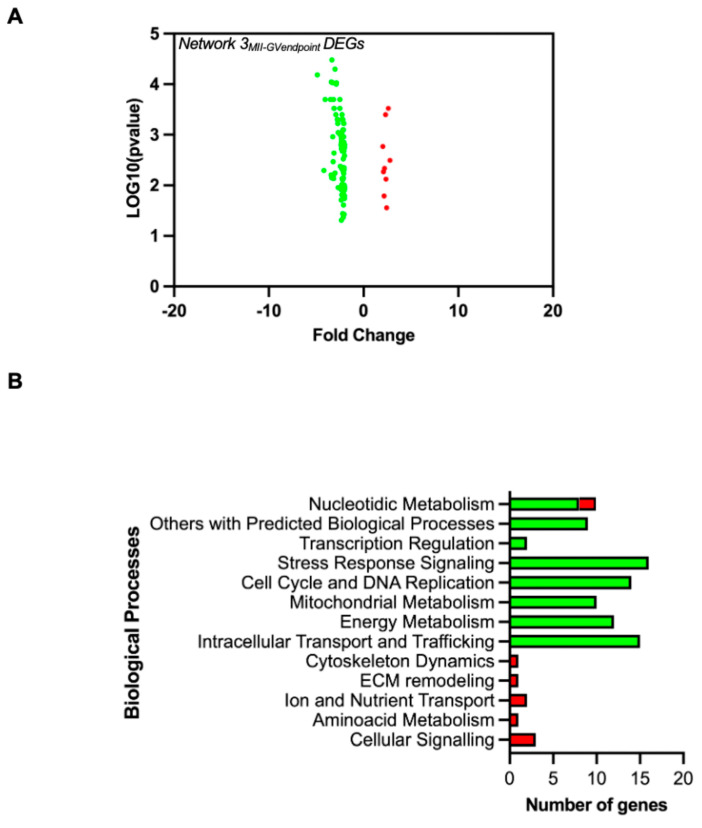
Network 3_MII-GVend-point_ clustering DEGs and classification for biological processes: (**A**) Volcano plot showing the upregulated and downregulated DEGs in Network 3_MII-GVend-point_. Red dots indicate upregulated genes, and the green dots indicate downregulated genes. (**B**) DEGs were retrieved in each biological process category. Red bars indicate upregulated genes, and the green bars indicate downregulated genes.

To delve into the details of the analysis, the maturation end-point transcriptome reveals 9 upregulated and 88 downregulated DEGs (Figure 4A and Table 2).

The Network 3_MII-GVend-point_ (MII end-point vs. GV end-point), built on the selected DEGs, recognized 97 nodes, 44 edges, and 56 connected components. The topological parameters listed in Supplementary Materials File S9 confirmed its scale-free nature.

Given the small DEG set, MCODE clustering was not performed; instead, DEGs were categorized by their associated biological processes.

As depicted in Figure 4B and detailed in Supplementary Materials File S10, upregulated DEGs in Networks 3_MII-GVend-point_ were predominantly enriched in metabolic and cellular homeostasis pathways—particularly amino acid metabolism, ion and nutrient transport, cellular signaling, ECM remodeling, and cytoskeleton dynamics—whereas downregulated DEGs mapped to energy, nucleotide, and mitochondrial metabolism, cell cycle control, transcriptional regulation, and DNA replication.

Driver genes were also selected in Network 3_MII-GVend-point_ by distinguishing between highly modulated and HUBs. No highly modulated DEGs were identified; however, 13 HUBs were filtered (Supplementary Materials File S11).

Specifically, the HUBs including seven DEGs involved in stress response and protein folding (*HSPA5*, *HSPA1A*, *HSPH1*, *HSPA6*, *DDIT3*, *HERPUD1*, and *DNAJB9*), two in cell cycle regulation (*MAD2L1*, *FBXO5*), three in DNA replication, repair, and chromatin modification (*GMNN*, *PRIM1*, *PHF5A*) and, finally, one related to ECM dynamics (*ITIH4*).

### 3.4. Validation of Predicted Driver Genes in Oocyte Maturation: qRT-PCR Confirmation and Literature Review

**Figure 5:**
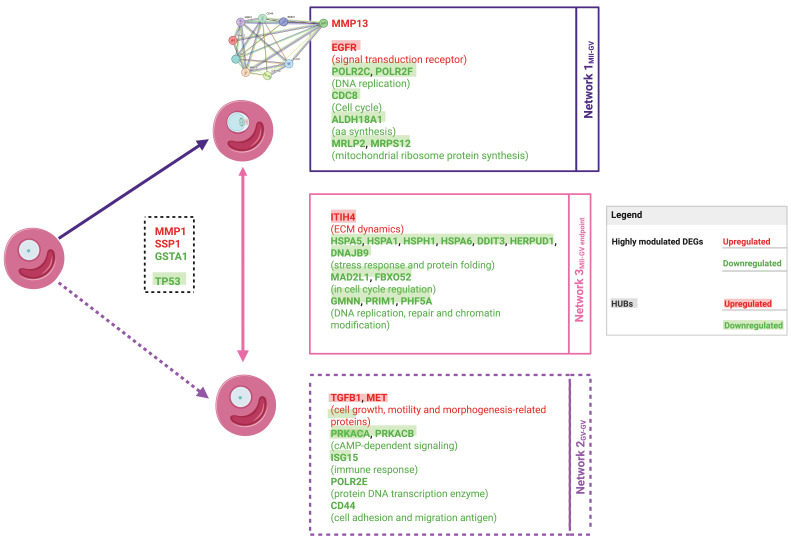
Graphical representation of all identified network drivers. The driver genes are classified according to their respective network of origin and their transcriptional modulations impacting the meiotic maturation process. The three networks are represented by distinct colors (Network 1_MII-GV_ in purple, Network 2_GV-GV_ in light blue, and Network 3_MII-GV end-point_ in orange). Transcriptional regulation levels are also color-coded, with red indicating upregulation and green indicating downregulation. Highly modulated DEGs belonging to KEGG categories are shown in bold. HUBs are underlined (red for upregulated, green for downregulated). Inside the dashed black rectangle, the drivers (highly modulated DEGs and HUBs) shared by both functional end-points (successful and unsuccessful maturation) are displayed, along with their respective transcriptional modulations. Created in Biorender. Peserico, A. (2025), https://www.biorender.com/.

Overall, the present transcriptomic and computational analysis identified genes with a potential regulatory role in the maturation process, also allowing the distinction of two different types of somatic biomarkers:

(I) Somatic markers differentiating between FWs before and after hCG-induced maturation (derived from Network 1_MII-GV_ and Network 2_GV-GV_).

(II) Somatic biomarkers of follicle leading to oocyte competence (derived from Network 3_MII-GV end-point_). Figure 5 provides an illustrated overview of all the identified drivers along with their modulations.

The identified candidate drivers were subjected to a final validation regarding their gene modulations found through array analysis (I), as well as a review of any reported roles in meiotic maturation described in the literature (II).

In more detail, gene expression data from the ovine array were validated through a qRT-PCR analysis of selected DEGs from each of the three networks. Specifically, highly modulated DEGs such as *MMP13* from Network 1_MII-GV_, the HUBs *CDCA8* from Network 1_MII-GV_, *TGFB1* and *ISG15* for Network 2_GV-GV_, and *HSPA6*, *GMNN*, and *ITIH4* for Network 3_MII-_GV end-point were selected (Supplementary Materials File S12).

Furthermore, the selected candidate genes were subjected to a comprehensive review of recent scientific literature to evaluate the latest insight into their biological roles in mediating the maturation process. The results of this literature review are summarized in Table 3. Notably, the biological reproductive role has been confirmed for some of the identified driver genes (15 out of 28), offering new insights for further in-depth investigations.

## 4. Discussion

This study provides new insights into the transcriptomic mechanisms regulating the FW of EAfs activated through hCG-mediated oocyte maturation. Using an FEO 3D culture system, somatic gene markers were identified that determine FW to be capable of supporting the maturation of high-quality oocytes from those that fail to trigger meiotic resumption.

A key merit of this study is the differentiation of molecular signatures governing meiosis resumption, alongside the identification of non-invasive somatic biomarkers predictive of oocyte competence. This is particularly critical for early antral follicles, the focus of our study. Although our FEO-IVM model yields high meiotic resumption rates (>65%) after hCG stimulation, the blastocyst formation rate remains low (~10%). Similar trends have been observed in other large mammals, where oocytes from smaller follicles show impaired cytoplasmic maturation and reduced cleavage and blastocyst yields versus those from larger follicles (ovine [94]; mouse [95]; bovine [96,97]). This limited developmental competence hinders the clinical use of early antral follicle-derived oocytes in ART and highlights the urgent need for non-invasive selection strategies. The somatic biomarkers identified here offer a means to enrich for oocytes from early follicles with true embryonic potential.

The identified DEGs fall into two major categories: (1) genes regulating meiosis resumption and (2) genes defining follicular signatures that support oocyte competence.

The first category—signing, signaling, and molecules required for meiosis resumption—was associated with the upregulation of ECM remodeling, relaxin, and hypoxia signaling pathways, while proliferative and metabolic pathways were downregulated.

Among the ECM-related genes, *MMP13* was highly modulated, reinforcing its established role in ECM breakdown and ovulation supported in different species [79,98,99,100,101,102,103,104,105,106]. Also, the epidermal *EGFR* appeared as a critical HUB gene in the EDC category, highlighting its regulatory role in ECM remodeling, which requires the regulation of somatic germinal coupling during ovulation [78].

The structural remodeling of the follicle required for ovulation also depends on the activation of the relaxin signaling pathway [107,108]. Accordingly, this study identified this pathway as upregulated in follicular cells enclosing the mature oocyte. Relaxin has been shown to act as a paracrine factor during the preovulatory phase, regulating collagenase activity [109], and key molecules of this pathway have been detected in the follicular fluid of women [110], equines [111], and pigs [112].

Hypoxia signaling was also pivotal in follicular maturation. *HIF-1α* activation was evident in FW supporting competent oocytes, reflecting its well-established role in facilitating the transition from periovulation to early corpus luteum differentiation under low oxygen conditions [113,114]. The HIF-1α-mediated regulation of ECM remodeling and glucose metabolism supports oocyte maturation, with inhibition of HIF-1α in vivo preventing oocyte release [115]. These findings further highlight hypoxia as a key regulator of successful follicular remodeling and oocyte competence.

The suppression of proliferative and metabolic pathways additionally supports the transition from granulosa cell proliferation to differentiation, thus reinforcing the metabolic quiescence hypothesis, which posits that successful meiosis resumption requires a coordinated metabolic rearrangement [116,117,118,119].

Conversely, in FW, after enclosing oocytes that failed to resume meiosis, *TGFB1* emerged as a critical HUB. TGFB1 is associated with meiotic arrest and follicular immaturity [120,121,122,123,124], suggesting that its upregulation may contribute to the failure of oocyte developmental progression.

The second category of DEGs was associated with follicular signatures supporting oocyte competence. Among these, *ITIH4* emerged as an upregulated HUB gene involved in angiogenesis, ECM stability, and ovulation-dependent inflammatory pathways. ITIH4 was notably upregulated in follicular fluid from follicles yielding mature oocytes, alongside key complement activation molecules [92,93], highlighting its role in the inflammatory response driving ovulation. Additionally, its contribution to angiogenesis and ECM stability is supported by its interaction with Von Willebrand factor and hyaluronan [125,126,127].

Moreover, the downregulation of cell cycle, DNA replication, and stress-related genes in the second DEG category suggests a lower cellular stress burden and improved genomic integrity. Accordingly, downregulated HUBs involved in cell cycle regulation and DNA replication (*MAD2L1*, *FBOX5*, *GMNN*, *PRIM1*, *PHF5A*) were found to be switched off when the differentiation shift occurs in response to hCG/LH [53]. Regarding the identified HUBs related to stress response and protein folding (*HSPA5*, *HSPA1A*, *HSPH1*, *HSPA6*, *DDIT3*, *HERPUD1*, and *DNAJB9*), although their role in the somatic compartment remains unclear [128], knockout models from the MGI database (see Table 3) highlight their reproductive functions.

## 5. Conclusions

This research advances our understanding by identifying novel DEGs associated with reproductive processes. Among these, some may represent novel regulators of follicular maturation and oocyte competence, opening new avenues for understanding follicular biology. Analyzing the genomic signature of FW with defined maturation outcomes might allow the integration of multi-omics approaches like proteomics and epigenomics, further elucidating regulatory networks governing oocyte competence.

A panel of non-invasive predictive biomarkers has been delineated: MMP13, EGFR, and ITIH4 are indicative of a follicular environment conducive to successful maturation, whereas TGFB1 marks failure. Assessing these genes in FW could refine oocyte selection in ART, improving fertilization rates and embryo quality. Additionally, TGFB1 may be a therapeutic target, as modulating TGF-β signaling could enhance oocyte maturation from EAfs.

Also, crucial transcriptomic signatures distinguishing FWs from EAfs releasing competent and incompetent oocytes were defined, highlighting ECM remodeling genes as key regulators of oocyte maturation. The discovery of previously uncharacterized DEGs offers new insights into follicular biology and ART advancements. To fully leverage these findings, validation across species and clinical settings is necessary. Future research should investigate dynamic gene expression changes during IVM to elucidate follicular activation and characterize differential signaling pathways and molecules in EAfs enclosing oocytes that progress to the blastocyst stage versus those from MII oocytes that do not, thereby enabling a more refined and accurate selection of gametes with improved success rates.

Additionally, functional studies, such as knockdown experiments, are essential to confirm the roles of newly identified DEGs in follicular maturation and oocyte competence. These efforts will help refine IVM protocols and improve oocyte selection strategies, despite the challenges posed by studying low-competent oocytes.

## Figures and Tables

**Table 1:** Comparative meiotic and developmental competence of FEO in vitro matured oocytes derived from early antral (EA) follicles.

**A**				
**Follicle Category**	**Healthy Oocytes** **(n°)**	**Oocyte Nuclear Stage**		
		**GV (%; SD)**	**GVBD/MI**	**MII**
EAf − hCG	48	100	--	--
EAf + hCG	236	11.0 ± 4.2	21.2 ± 5.5	67.8 ± 6.3
**B**				
	**Healthy Oocytes** **(n°)**	**Post-Fertilization Embryo Development**		
		**Uncleaved** **(%; SD)**	**Fertilization Rate** **(%; SD)**	**Blastocyst Rate** **(%; SD)**
EAf	160	32 ± 5.2	58.2 ± 5.5	9.9 ± 3.3

Comparison of meiotic and developmental competencies of EAf-enclosed oocytes. Table 1A FEO in vitro maturation was performed according to a validated method [1] by exposing EAfs (400 ± 26 µm diameter size) to hCG (25 IU/mL) in the presence of ovarian surface epithelial cells. Follicular cells (FW) from EAfs were collected at the end of the 24 h maturation window, according to the nuclear stage of the enclosed oocytes. The FWs of high-quality MII (only those reaching the blastocyst stage) were finally selected after IVF for the transcriptomic analysis. Table 1B shows the developmental competence of MII oocytes after IVF, tested to collect FWs for analysis. The fertilization rate was calculated based on the number of cleaved embryos at day 2. The blastocyst rate was calculated at day 8 on the cleaved embryo recorded on day 2. Data are expressed as the mean value of three independent biological replicates.

**Table 2:** Tree pairwise comparisons of DEGs.

Pairwise Comparison	Generated Network	Total Number of Genes	Genes passed Filter Criteria (DEG)	Genes with No Changes	Upregulated	Downregulated
Pairwise 1	Network 1_MII-GV_	22,141	2526 (11.4%)	19,615 (88.6%)	854 (33.8%)	1672 (66.2%)
Pairwise 2	Network 2_GV-GV_	22,141	1998 (9%)	20,143 (91%)	878 (43.9%)	1120 (56%)
Pairwise 3	Network 3_MII-GV end-point_	22,141	97 (0.44%)	22,044 (99.6%)	9 (9.3%)	88 (90.7%)

The filtering criteria for identifying DEGs using GeneChip array analysis with Affimetrix include a fold change greater than 2 and a *p*-value less than 0.05. DEGs obtained from each pairwise comparison were used as input to build the corresponding gene-interaction network through systems biology workflows, as detailed in Section 3.2 and Section 3.3.

**Table 3:** Literature annotations on the female reproductive role of the identified candidate driver genes.

	Driver Gene Name	Network	Mcode	DEG Fold Change	*p*-Value	Female Fertility Effects	
						Knockout/Mutant Mice	Effects Related to Maturation
AIM 1: Somatic Markers Distinguishing FW Pre- and Post-hCG Maturation	*CDCA8*	1	4	−2.09	0.0014	N/A	N/A any FWs role Role in oocyte: it regulates meiotic spindle assembly and chromosome segregation during human oocyte meiosis [76]
	*EGFR*	1	8	2.63	0.0001	Mutant mice (Egfr^wa2^/Egfr^Wa5^) show infertility (J:92308 from the MGI database.	EGFR activation is crucial for hCG-induced progesterone production in human granulosa cells, with amphiregulin (AREG) mediating this effect by upregulating StAR expression [77]; EGFR signaling in granulosa cells at ovulation triggers filopodia retraction, uncoupling germ and somatic cells [78]
	*MMP13*	1	7	21.18	3.37 × 10^−6^	N/A	Gonadotropin Surge-Induced MMP-13 mRNA and protein in bovine preovulatory follicles [79]. Upregulation of MMP13, together with M2 macrophage polarization by the antifibrotic drug BGP-15, facilitated ovulation in old and obese mice [80]
	*MMP1*	1; 2	8; 2	73.12; 106.96	2.24 × 10^−8^ 6.21 × 10^−7^	N/A	Affected reproductive processes, including ovulation and folliculogenesis in chickens, drosophila, macaque, and humans [81,82,83,84,85,86]
	*ISG15*	2	1	−2.97	0.0009	Knockout in mice causes hyperfertility along with sensitive ovarian responses to gonadotropin, such as increases in cumulus expansion and ovulation rate [87].	N/A
	*SPP1*	1; 2	7; 2	51.92; 38.49	3.67 × 10^−10^ 1.27 × 10^−8^	N/A	SPP1/Opn upregulation in preovulatory granulosa cells, triggered by gonadotropin via EGFR signaling, boosts progesterone synthesis and VEGF expression during the early luteal phase [88]. In antral follicles, SPP1 is responsible for immune processes leading to ovarian follicular atresia [89]. SPP1 is upregulated in response to hormonal cues, particularly during the periovulatory phase, where it contributes to follicular rupture and corpus luteum formation [90]
	*TGFB1*	2	2	2.23	0.0009	Knock out (Tgfb1tm1(Tgfb3)Kul/Tgfb1tm1(Tgfb3)Kul) in mice causes infertility (J:204892 from MGI database).	N/A
AIM 2: Somatic Biomarkers of Follicular and Enclosed Oocyte Competence	*HSPA5*	3	/	−3.37	0.0071	Knock-out phenotype observed for the master regulator of HSP proteins, HSF1. HSF1 knock out (Hsf1tm1Ijb/Hsf1tm1Ijb) in female mice causes infertility (J:58383, J:65267); abnormal female meiosis and abnormal meiotic spindle assembly checkpoint (J:175085).	N/A
	*HSPA1A*	3	/	−3.37	0.0071		N/A
	*HSPH1*	3	/	−2.34	0.0019		N/A
	*HSPA6*	3	/	−4.19	0.0051		N/A
	*MAD2L1*	3	/	−3.16	0.0074	Mutant mice (Mad2l2repro22/Mad2l2repro22) show infertility (J:92463).	N/A
	*GMNN*	3	/	−2.21	0.0078	Oocyte-specific disruption of geminin (Gdf9-Cre Gmnn fl/fl) results in low fertility in mice. Even though there was no evident anomaly of oogenesis, oocyte meiotic maturation, natural ovulation, or fertilization, early embryo development and implantation were impaired (MGI Database).	N/A
	*FBXO5*	3	/	−2.1	0.0006	No role in FWs Role in oocyte: During oocyte maturation, it plays a role in meiosis through the inactivation of the APC-FZR1 complex. Inhibits APC through RPS6KA2 interaction that increases FBXO5 affinity for CDC20, leading to the metaphase arrest of the second meiotic division before fertilization [91].	N/A
	*ITIH4*	3	/	2.09	0.0054	N/A	Involved in the stabilization of the extracellular matrix and inflammatory processes. Found to be upregulated as a protein in FF from mature vs. immature oocytes (bovine) along with other proteins involved in complement activation (ITIH4, AHSG, FN1, HP) [92]. This inflammatory pathway may facilitate the local physiological and inflammatory reaction of ovulation [93].

Summary of annotated data for driver genes, for which literature information has been gathered regarding their roles in maturation. Other descriptive details are summarized, including gene name, associated network, MCODE cluster, DEG fold change, *p*-value, and role in maturation. These integrated annotations provide the mechanistic rationale underpinning the selection of every driver gene and pathway. N/A refers to “Not annotated”. Phenotypes from mutant mice were checked using the Mouse Genome Database (MGI; https://www.informatics.jax.org, accessed on 2 May 2024) and PubMed.

## Data Availability

All data generated or analyzed during this study are included in this published article and its Supplementary Materials Files.

## Supplementary Materials

The following supporting information can be downloaded at: https://www.mdpi.com/article/10.3390/cells14100704/s1, File S1: Main Topological Parameters Assessed in the Present Study by Cytoscape Network Analyzer. File S2: List of centrality coefficients calculated in the present study by CytoHubba plugin of Cytoscape. File S3: Sequence of primers used for qPCR validation. File S4: Network 1_MII-GV_ and Network 2_GV-GV_ topological parameters. File S5: List of DEGs, clustering into MCODE modules and identification of the largest KEGG pathway categories in Network 1_MII-GV_ and Network 2_GV-GV_. File S6: MMP13 in the MCODE cluster 7, connects with genes involved in ECM remodeling. The graphic depicts the PPI String computed interaction of *MMP13* within genes of cluster 7 of Network 1. File S7: Network 1_MII-GV_ and Network 2_GV-GV_ centrality parameters. File S8: Network 1_MII-GV_ and Network 2_GV-GV_ top 10 DEGs defined on each centrality coefficient score. File S9: Network 3_MII-GVend-point_ topological parameters. The table displays the computed topological parameters. File S10: Classification of DEGs in Network 3_MII-GVend-point_ based on the biological processes in which they are involved. File S11: Centrality parameters computed by CytoHUBba for Network 3_MII-GVend-point_. Sheet 1 contains 12 centrality coefficients computed for each DEG of Network 3_MII-GVend-point_. File S12: Real time qPCR validation of GeneChip data, related to selected DEGs.
