# Supplementary material for: Transcriptomic Profile of Early Antral Follicles: Predictive Somatic Gene Markers of Oocyte Maturation Outcome
Source: Cells. 2025 May 12;14(10):704. doi: 10.3390/cells14100704 (PMC12110445; doi:10.3390/cells14100704)
Supplement: Supplementary file 1 [file cells-14-00704-s001.zip › ADDITIONAL FILES Cells revised/Additional File S1.pdf]

Additional File S1

Parameter	Definition
Number of nodes	Total number of molecules involved
Number of edges	Total number of interactions found
Averaged number of neighbors	Mean number of connections of each node
Network diameter	Represents the shortest path between any two nodes in a network. Calculated using the shortest path betweenness algorithm, which employs Breadth-First Search.
Characteristic path length	Expected distance between two connected nodes
Clustering coefficient	It is a measure of how the nodes tend to form clusters. Calculated as CI = 2nI/kI(kI–1), where nI is the number of links connecting the kI neighbors of node I to each other.
Connected components	The maximal set of nodes such that each pair of nodes is connected by a path.

Main Topological Parameters Assessed in the Present Study by Cytoscape Network Analyzer
