# Supplementary material for: Transcriptomic Profile of Early Antral Follicles: Predictive Somatic Gene Markers of Oocyte Maturation Outcome
Source: Cells. 2025 May 12;14(10):704. doi: 10.3390/cells14100704 (PMC12110445; doi:10.3390/cells14100704)
Supplement: Supplementary file 1 [file cells-14-00704-s001.zip › ADDITIONAL FILES Cells revised/Additional File S10.pdf]

Additional File S10

DEG NAME	DESCRIPTION	FOLD CHANGE	P-VALUE	BIOLOGICAL PROCESS
TOMM20	Translocase Of Outer Mitochondrial Membrane 20	-2,08	0,0014	Intracellular Transport and Trafficking
TMED1	Transmembrane P24 Trafficking Protein 1	-2,07	0,0021	
TMEM70	Transmembrane Protein 70	-2,15	0,003	
TMEM45A	Transmembrane Protein 45°	-2,38	0,0016	
RAB32	RAB32, Member RAS Oncogene Family	-2,31	0,0009	
RAB9A	RAB9A, Member RAS Oncogene Family	-2,46	0,0042	
SLC25A33	Solute Carrier Family 25 Member 33	-3,42	9,06E-05	
SLC26A11	Solute Carrier Family 26 Member 11	-2,08	0,0006	
SLC35G1	Solute Carrier Family 35 Member G1	-2,14	0,0054	
AKAP12	A Kinase Anchoring Protein 12	-2,29	0,0097	
CLDN5	Claudin 5	-2,02	0,0016	
TES	Testin LIM Domain Protein	-2,34	0,0012	
BLOC1S2	Biogenesis Of Lysosomal Organelles Complex 1 Subunit 2	-2,11	0,0101	
TM4SF18	Transmembrane 4 L Six Family Member 18	-2,35	0,0123	
VLDLR	Very Low Density Lipoprotein Receptor	-2,72	0,0006	
KCTD7	Potassium Channel Tetramerization Domain Containing 7	-2,49	0,0002	
HSD17B1	Hydroxysteroid 17Beta Dehydrogenase 1	-3,34	3,29E-05	Energy Metabolism
CMBL	CarboxymethylenebutenolidaseLike	-2,28	0,0005	
EMC7	ER Membrane Protein Complex Subunit 7	-2,03	0,0125	
IDI1	IsopentenylDiphosphate Delta Isomerase 1	-2,86	9,43E-05	
CA9	Carbonic Anhydrase 9	-3,01	5,05E-05	
ERO1L	ERO1Like Protein Alpha	-3,21	0,0034	
PHGDH	Phosphoglycerate Dehydrogenase	-2,74	0,0005	
ACYP1	Acylphosphatase 1	-2,16	0,0122	
ACYP2	Acylphosphatase 2	-2,15	0,0428	
EGLN3	Egl9 Family Hypoxia Inducible Factor 3	-2,08	0,012	
ISYNA1	Inositol3Phosphate Synthase 1	-2,68	0,0009	
PCED1B	PCEsterase Domain Containing 1B	-2,12	0,0008	

Additional File S10

ATP5S	ATP Synthase Membrane Subunit S	-2,34	0,0491	Mitochondrial Metabolism
NDUFB11	NADH:Ubiquinone Oxidoreductase Subunit B11	-2,13	0,0072	
MRPL45	Mitochondrial Ribosomal Protein L45	-2,77	0,0005	
MRPL53	Mitochondrial Ribosomal Protein L53	-2,05	0,0045	
MRPS36	Mitochondrial Ribosomal Protein S36	-2,68	0,0111	
CYP51A1	Cytochrome P450 Family 51 Subfamily A Member 1	-3,25	0,0011	
ECI1	EnoylCoA Hydratase 1	-3,17	0,0002	
MSMO1	Methylsterol Monooxygenase 1	-4,05	0,0002	
CIART	Circadian Associated Repressor Of Transcription	-2,23	0,0005	
TLL2	TolloidLike Protein 2	-2,02	0,0104	
PRIM1	DNA Primase Subunit 1	-2,3	0,0047	Cell cycle and DNA Replication
RFC4	Replication Factor C Subunit 4	-2,14	0,0047	
GMNN	Geminin DNA Replication Inhibitor	-2,21	0,0078	
DDIT4	DNA Damage Inducible Transcript 4	-2,88	0,0001	
DHRS1	Dehydrogenase/Reductase 1	-2	0,0114	
HERPUD1	HomocysteineInducible ER Protein with UbiquitinLike Domain 1	-2,92	0,0004	
MAD2L1	Mitotic Arrest Deficient 2 Like 1	-3,16	0,0074	
ANKRD37	Ankyrin Repeat Domain 37	-3,11	0,0003	
FBXO5	FBox Protein 5	-2,1	0,0006	
NIPSNAP3A	Nipsnap Homolog 3A	-3,02	0,0057	
CENPW	Centromere Protein W	-2,2	0,0005	
BTG3	BCell Translocation Gene 3	-2,02	0,0162	
NCAPG	NonSMC Condensin I Complex Subunit G	-2,18	0,0008	
LOC114110673	Uncharacterized LOC114110673	-4,88	6,54E-05	
PTGR1	Prostaglandin Reductase 1	-2,25	0,0021	Nucleic metabolism
PHF5A	PHD Finger Protein 5°	-2,17	0,015	
CEBPG	CCAAT/Enhancer Binding Protein Gamma	-2,37	0,001	
DNAJB9	DnaJ Heat Shock Protein Family (HSP40) Member B9	-2,37	0,0195	

Additional File S10

SNRNP25	Small Nuclear Ribonucleoprotein U11/U12 Subunit 25	-2,06	0,0049	
LSM3	LSM3 Homolog, U6 Small Nuclear RNA and mRNA Degradation Associated	-2,27	0,0074	
PTTG1IP	Pituitary TumorTransforming 1 Interacting Protein	-2,12	0,0151	
DDIAS	DNA Damage Induced Apoptosis Suppressor	-2,35	0,0014	
TRMT10C	TRNA Methyltransferase 10C, Mitochondrial RNase P Subunit	-2,12	0,0246	
DDIT3	DNA Damage Inducible Transcript 3	-2	0,0016	Stress response signaling
HSPA1A	Heat Shock Protein Family A (Hsp70) Member 1A	-3,37	0,0071	
HSPA5	Heat Shock Protein Family A (Hsp70) Member 5	-3,5	0,0002	
HSPA6	Heat Shock Protein Family A (Hsp70) Member 6	-4,19	0,0051	
HSPH1	Heat Shock Protein Family H (Hsp105) Member 1	-2,34	0,0019	
GADD45G	Growth Arrest and DNA Damage Inducible Gamma	-3,44	0,0062	
ARG2	Arginase 2	-3,12	0,0023	
ADIRF	Adipogenesis Regulatory Factor	-3,13	0,0003	
IER3IP1	Immediate Early Response 3 Interacting Protein 1	-2,26	0,0015	
RGCC	Regulator Of Cell Cycle	-3,27	9,35E-05	
SDE2	SDE2 Telomere Maintenance Homolog	-2,11	0,0011	
LY96	Lymphocyte Antigen 96	-2,01	0,0119	
TNFRSF12A	TNF Receptor Superfamily Member 12A	-2,01	0,0018	
OSER1	Oxidative Stress Responsive Serine Rich 1	-2,27	0,0004	
SELK	Selenoprotein K	-2,31	0,0154	
C1D	C1D Nuclear Receptor Corepressor	-2,05	0,0375	
ZNF791	Zinc Finger Protein 791	-2,1	0,0058	Transcription Regulation
BEND5	BEN Domain Containing 5	-2,21	0,0044	
BEX5	Brain Expressed XLinked 5	-2,18	0,0366	Biological process need to be annotated
CRISP2	CysteineRich Secretory Protein 2	-2,01	0,0026	
EBPL	Emopamil Binding ProteinLike	-2,02	0,0183	
GCOM1	GRINL1A Complex Locus 1	-2,06	0,0018	
LOC101101998	Uncharacterized LOC101101998	-2,47	0,001	

Additional File S10

LOC101115217	Uncharacterized LOC101115217	-3,19	0,0065	
LOC101118478	Uncharacterized LOC101118478	-2,22	0,0362	
LOC101121364	Uncharacterized LOC101121364	-2,49	0,0003	
SMNDC1	Survival Of Motor Neuron Domain Containing 1	-2,06	0,0099	
AMMECR1	AMMECR1 Chromosome Region 1	2,22	0,0004	Cellular Signaling
RASSF3 (synonym RASSF5)	Ras Association Domain Family Member 3	2,78	0,0003	
RGS6	Regulator Of GProtein Signaling 6	2,43	0,0046	
IGFBP5	Insulin Like Growth Factor Binding Protein 5	2,78	0,0032	
AHCYL2	SAdenosylhomocysteine HydrolaseLike 2	2,17	0,0278	Aminoacid Metabolism
SLC43A2	Solute Carrier Family 43 Member 2	2,03	0,0162	Ion and Nutrient Transport
LOC101116002	Chloride Channel CLIC Like 1	2.36	0.0075	
ITIH4	InterAlphaTrypsin Inhibitor Heavy Chain Family Member 4	2,36	0,0054	ECM Remodeling
DIAPH2	Diaphanous Related Formin 2	2,32	0,0017	Cytoskeleton Dynamics

Classification of DEGs in Network $3_{MII\text{-}GVendpoint}$ based on the biological processes in which they are involved. Attributes, including fold change, p-value, and related biological process, are reported for each DEG.
