# Supplementary material for: Transcriptomic Profile of Early Antral Follicles: Predictive Somatic Gene Markers of Oocyte Maturation Outcome
Source: Cells. 2025 May 12;14(10):704. doi: 10.3390/cells14100704 (PMC12110445; doi:10.3390/cells14100704)
Supplement: Supplementary file 1 [file cells-14-00704-s001.zip › ADDITIONAL FILES Cells revised/Additional File S12.pdf]

Additional File S12

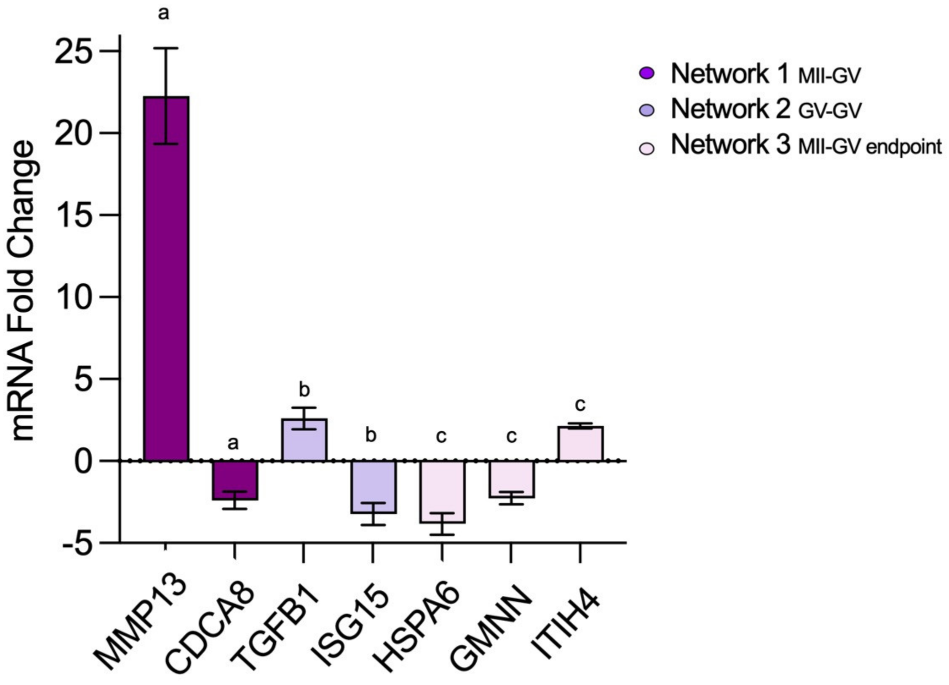

Real time qPCR validation of GeneChip data, related to selected DEGs. Real-Time qPCR validation of most representative genes. Fold-changes are related to the mean ± SD of 2^- ΔΔCt values obtained in n = 3 independent experiments. For Network $1_{MII\text{-}GV}$, the significance of *MMP13* and *CDCA8* data related FCs enclosing MII oocytes at the end of FEO ($p < 0.05$) was statistically analyzed vs. FCs enclosing GV oocytes as the starting point of FEO and indicated with (a). For Network $2_{GV\text{-}GV}$, the significance of *TGFB1* and *ISG15* data related to FCs enclosing GV oocytes at the end of FEO ($p < 0.05$) was statistically analyzed vs. FCs enclosing GV oocytes as the starting point of FEO and indicated with (b). For Network $3_{MII\text{-}GV\ endpoint}$, the significance of *HSPA6*, *GMNN* and *ITIH4* data related to FCs enclosing MII oocytes at the end of FEO ($p < 0.05$) was statistically analyzed vs. FCs enclosing GV oocytes at the end of FEO and indicated with (c).

Classification of DEGs in Network $3_{MII\text{-}GVendpoint}$ based on the biological processes in which they are involved. Attributes, including fold change, p-value, and related biological process, are reported for each DEG.
