# Supplementary material for: Transcriptomic Profile of Early Antral Follicles: Predictive Somatic Gene Markers of Oocyte Maturation Outcome
Source: Cells. 2025 May 12;14(10):704. doi: 10.3390/cells14100704 (PMC12110445; doi:10.3390/cells14100704)
Supplement: Supplementary file 1 [file cells-14-00704-s001.zip › ADDITIONAL FILES Cells revised/Additional File S2.pdf]

Additional File S2

Coefficient name	Definition
Closeness (Clo)	It quantifies how close a node is to all other nodes in the network. $$Clo(v) = \sum_{w \in V} \frac{1}{dist(v,w)}$$ Where:<br>$C(v)$ is the closeness centrality of node v.<br>N is the total number of nodes in the network.<br>$D(v,u)$ is the shortest path distance between node v and node u.
Degree	The degree counts how many neighbors (or direct connections) node v has in the network. $$Deg(v) = \|N(v)\|$$ Where:<br>$N(v)$ is the degree of node v
Maximal Clique Centrality (MCC)	It is a measure that identifies nodes based on the size of the largest clique they are part of. $$MCC(v) = \frac{\|C(v)\|}{\|N(v)\|}$$ Where:<br>$\|C(v)\|$ is the size of the largest clique that includes node v<br>$\|N(v)\|$ is the number of neighbors of node v
Radiality (Rad)	The radiality of a node is the average distance from that node to all other nodes. $$Rad(v) = \frac{\|V(C(v))\|}{\|V\|} \times \frac{\sum w \in C(v)(\Delta_{C(v)} + 1 - dist(v,w))}{\max\{dist(v,w): w \in C(v)\}}$$ Where $\Delta_{C(v)}$ is the maximum distance between any two vertices of the component $C(v)$.
Stress (Str)	This centrality is a measure based on the total number of shortest paths that pass through a node. $$Str(v) = \sum s \neq t \neq v \in C(v)\, \sigma_{st}(v)$$ Where:<br>$\sigma_{st}(v)$ is the number of shortest paths from node s to node t.
Maximum Neighborhood Component (MCN)	This measure is based on the maximum size of the cliques a node belongs to. It measures the participation of a node in the largest cliques in the network. $$MNC(v) = \max_{C \in C} \|C\|$$ Where:<br>C is the set of all cliques containing node v<br>| C | is the size of the clique C
Density of Maximum Neighborhood Component (DNMC)	This coefficient calculates the density of the largest clique in which a node participates. The density is defined as the ratio of the number of edges within the clique to the maximum possible number of edges. $$DMNC(v) = \|E(MC(v))\|/\|V(MC(v))\|$$ Where:

	$\lvert E(MC(v))\rvert$ is the number of edges in the clique MC that node v is part of<br>$\lvert MC(v)\rvert$ is the size of the clique $MC(v)$
Betweenness (BC)	Measures the extent to which a node lies on the shortest paths between other nodes in the network.<br>$$BC(v) = \sum s \neq t \neq v \in C(v) \frac{\sigma_{st}(v)}{\sigma_{st}}$$<br>Where:<br>σ_{st} is the number of shortest paths from node s to node t.
Clustering Coefficient (CC)	The formula counts the actual edges between neighbors of node v and compares it to the maximum number of possible edges between them.<br>$$C(v) = \frac{2 \cdot E(v)}{k_v(k_v - 1)}$$<br>Where:<br>$E(v)$is the number of edges between the neighbors of node v<br>$k_v$is the number of neighbors of node v (degree of node v)
EcCentricity (EC)	It determines how fare a node is from the most distant node in the network. $EC(v) = \frac{\lvert V(C(v))\rvert}{\lvert V\rvert} \times \frac{1}{\max\{dist(v,w):w \in C(v)\}}$<br><br>Where:<br>$d(v, u)$ is the shortest path distance between node v and node u.<br>Max represents the longest shortest path from v to any other node in the network.
Bottleneck (BN)	This coefficient identifies nodes whose removal would disconnect parts of the network, potentially increasing the diameter of the network.<br>$$BN(v) = \sum_{s \in V} P_s(v)$$<br>Where:<br>$P_s(v)$ =1 if more that $\lvert V(T_s)\rvert/4$ paths from node s to other nodes in (T_s) meet at the vertex v
Edge Percolated Component (EPC)	Measures the size of the connected component that remains after removing the edges incident to the node.<br>$$EPC(v) = \frac{1}{\lvert V\rvert} \sum_{k=1}^{1000} \sum_{t \in V} \delta_{vt}^{k}$$<br>Where:<br>$\lvert V\rvert$is the total number of nodes in the network<br>$\sum_{k=1}^{1000}$ is the summation over 1000 iterations<br>$\sum t \in V$ Is the summation over all nodes t in the network.<br>δ_{vt}^{k}Is the key function that is being summed over

List of centrality coefficients calculated in the present study by CytoHubba plugin of Cytoscape.
