# Supplementary material for: Transcriptomic Profile of Early Antral Follicles: Predictive Somatic Gene Markers of Oocyte Maturation Outcome
Source: Cells. 2025 May 12;14(10):704. doi: 10.3390/cells14100704 (PMC12110445; doi:10.3390/cells14100704)
Supplement: Supplementary file 1 [file cells-14-00704-s001.zip › ADDITIONAL FILES Cells revised/Additional File S3.pdf]

Additional File S3

**Gene**	**Forward (5’-3’)**	**Reverse (5’-3’)**
MMP13	TCCAGTTTGCAGAGAGCTACC	ATCACAGAGCTTGCTGCACT
CDCA8	TCTTAGGCTCGCCTTTTGGG	TGCTGCTAGGTAAGCAGGAG
TGFB1	ACAATTCCTGGCGCTACCTC	ACCCGTTGATGTCCACTTGA
ISG15	GAGACTGGCACCAGAACCC	ACTGCTTCAGCTCGGATACC
HSPA6	ACGGGAAGGATTTGAGCAGG	GGTCCGAGCACAGTTCTTCA
GMNN	GATGAAATTGCCCGCCTGAA	GTATCCTCAGCAGTTGCCTGT
ITIH4	TACGTCCTGGTGGAGCATCT	CACCTCAACCCCTTGGTCAG
GAPDH	TCGGAGTGAACGGATTTGGC	CCGTTCTCTGCCTTGACTGT
YWHAZ	AGACGGAAGGTGCTGAGAAA	CGTTGGGGATCAAGAACTTT-

Sequence of primers used for qPCR validation
