# Supplementary material for: Transcriptomic Profile of Early Antral Follicles: Predictive Somatic Gene Markers of Oocyte Maturation Outcome
Source: Cells. 2025 May 12;14(10):704. doi: 10.3390/cells14100704 (PMC12110445; doi:10.3390/cells14100704)
Supplement: Supplementary file 1 [file cells-14-00704-s001.zip › ADDITIONAL FILES Cells revised/Additional File S4.pdf]

Additional File S4

**Statistics**	**Network 1_{MII-GV}**	**Network 2_{GV-GV}**
Number of nodes	2.071	1.562
Number of edges	15.712	6.546
Avg. number of neighbors	15.878	8.923
Network diameter	8	9
Characteristic path length	3.503	3.930
Clustering coefficient	0.238	0.247
Connected components	92	95

Network 1_{MII-GV} and Network 2_{GV-GV} topological parameters.
