# Supplementary material for: Transcriptomic Profile of Early Antral Follicles: Predictive Somatic Gene Markers of Oocyte Maturation Outcome
Source: Cells. 2025 May 12;14(10):704. doi: 10.3390/cells14100704 (PMC12110445; doi:10.3390/cells14100704)
Supplement: Supplementary file 1 [file cells-14-00704-s001.zip › ADDITIONAL FILES Cells revised/Additional File S6.pdf]

Additional File S6

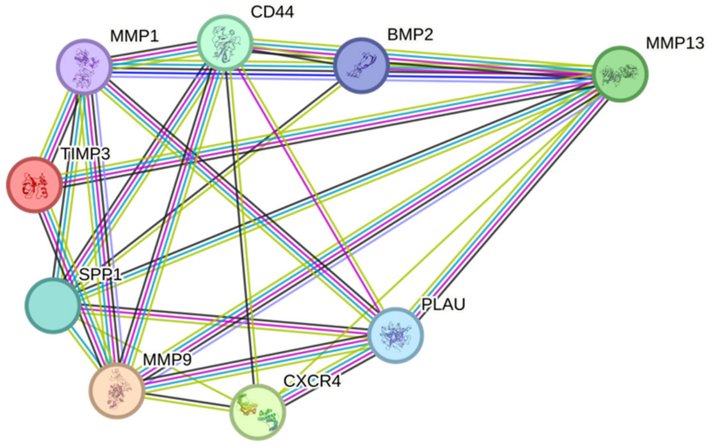

MMP13 in the MCODE cluster 7, connects with genes involved in ECM remodeling. The graphic depicts the PPI String computed interaction of *MMP13* within genes of cluster 7 of Network 1.
