# Supplementary material for: Transcriptomic Profile of Early Antral Follicles: Predictive Somatic Gene Markers of Oocyte Maturation Outcome
Source: Cells. 2025 May 12;14(10):704. doi: 10.3390/cells14100704 (PMC12110445; doi:10.3390/cells14100704)
Supplement: Supplementary file 1 [file cells-14-00704-s001.zip › ADDITIONAL FILES Cells revised/Additional File S9.pdf]

Additional File S9

**Statistics**	**Network** $3_{MII\text{-}GVendpoint}$
Number of nodes	97
Number of edges	44
Avg. number of neighbors	3000
Network diameter	5
Characteristic path lenght	2.242
Clustering coefficient	0.342
Connected components	56

Network $\mathbf{3_{MII\text{-}GVendpoint}}$ **topological parameters.** The table displays the computed topological parameters.
